# Human Cerebral Blood Flow Activity with Diurnal Variation Differentially Correlated with the Suprachiasmatic Nucleus

**DOI:** 10.1523/JNEUROSCI.0729-25.2026

**Published:** 2026-03-12

**Authors:** Akitoshi Ogawa, Satoshi Oka, Takahiro Osada, Masaki Tanaka, Weihang Chen, Koji Kamagata, Shigeki Aoki, Takahiro J. Nakamura, Seiki Konishi

**Affiliations:** ^1^Department of Neurophysiology, Juntendo University School of Medicine, Tokyo 113-8421, Japan; ^2^Department of Economics, Sapporo Gakuin University, Sapporo 004-8666, Japan; ^3^Department of Radiology, Juntendo University School of Medicine, Tokyo 113-8421, Japan; ^4^Laboratory of Animal Physiology, School of Agriculture, Meiji University, Kanagawa 214-8571, Japan; ^5^Sportology Center, Juntendo University School of Medicine, Tokyo 113-8421, Japan; ^6^Research Institute for Diseases of Old Age, Juntendo University School of Medicine, Tokyo 113-8421, Japan; ^7^Advanced Research Institute for Health Science, Juntendo University School of Medicine, Tokyo 113-8421, Japan

**Keywords:** cerebral blood flow, functional connectivity, functional magnetic resonance imaging, hippocampus, human, primary visual area

## Abstract

The human circadian rhythm is controlled by central and peripheral clocks, primarily by the central clock in the suprachiasmatic nucleus (SCN). We investigated the diurnal variation of basic metabolism in the human cerebrum by measuring human baseline cerebral activity at rest contrasted with the SCN baseline activity. To this end, we utilized magnetic resonance imaging perfusion data of cerebral blood flow (CBF; *N* = 27, including both sexes), where each participant was scanned four times a day at 6 h intervals (18:00, 24:00, 6:00, and 12:00 local time). Similarly to the SCN exhibiting higher CBF activity at noon, we observed a consistent temporal activity pattern in the brain regions, including the limbic (cingulate, insular, and temporopolar) and sensorimotor (visual and somatosensory/motor) areas. In contrast, the hippocampus showed higher activity at midnight and lower activity at noon. To examine the functional interaction between the SCN and the cerebral regions showing diurnal variation, we calculated the resting-state functional connectivity using the database of the Human Connectome Project (*N* = 164, including both sexes). Notably, the hippocampus demonstrated greater functional connectivity with the SCN than the other regions. These results suggest that cerebral regions exhibit differential patterns of diurnal variation associated with their functional connectivity with the SCN.

## Significance Statement

The human circadian rhythm is regulated primarily by the suprachiasmatic nucleus (SCN) and peripheral clocks. This study examined diurnal variations in human cerebral activity by measuring baseline cerebral blood flow at rest using magnetic resonance imaging. Participants were scanned four times a day (18:00, 24:00, 6:00, and 12:00). Similar to the SCN, cerebral regions in the limbic and sensorimotor areas showed higher activity at noon. However, the hippocampus exhibited peak activity at midnight and lower activity at noon. Functional connectivity analysis revealed stronger SCN–hippocampus connectivity than the other cerebral regions. These findings suggest that brain areas (i.e., the hippocampus and the limbic and sensorimotor areas) show distinct diurnal activity patterns linked to their functional connectivity with the SCN.

## Introduction

Circadian rhythms regulate physiology and behavior according to the 24 h cycle of day and night (Hastings et al., 2018). Circadian disruption is related to diseases affecting various human systems, including the autonomic and endocrine systems (Cai et al., 2020; Fishbein et al., 2021). The suprachiasmatic nucleus (SCN) in the hypothalamus is the central clock for circadian rhythms in mammals, transmitting timing signals to other parts of the central nervous system (Moore and Eichler, 1972; Stephan and Zucker, 1972; Inouye and Kawamura, 1979, 1982; Sumova et al., 1995; Welsh et al., 1995, 2010; Nagano et al., 2003; Yamaguchi et al., 2003; Saper et al., 2005a,b; Enoki et al., 2012; Musiek and Holtzman, 2016; Hastings et al., 2018). Human postmortem studies have shown diurnal cycles of neuropeptides in the SCN (Hofman and Swaab, 1993, 1994; Hofman, 2000). However, noninvasive measurement of human SCN activity has been challenging due to its small size. Recent advances in high-resolution functional magnetic resonance imaging (MRI) have enabled the detection of SCN activity in vivo (Schoonderwoerd et al., 2022; Campbell et al., 2024), revealing that the SCN exhibits higher activity in daylight time and lower activity at night and in the early morning (Oka et al., 2024).

Beyond the SCN, diurnal variation also occurs in the mammalian cerebrum. Clock gene expression has been observed in the cerebral cortex as well as the SCN (Yang et al., 2007; Franken and Dijk, 2009; Cermakian et al., 2011; Li et al., 2013; Lim et al., 2013; Rath et al., 2013, 2014; Chen et al., 2016; Bering et al., 2017, 2023; Liu et al., 2019) and is associated with circadian phenotypes, homeostatic regulation of sleep, and cognitive performance (Dewandre et al., 2018). In addition to gene expression, other physiological variables, such as the regional cerebral metabolic rate of glucose (Germain et al., 2007) and brain volume (Nakamura et al., 2015; Kim et al., 2024), fluctuate throughout the day. The SCN activity influences motor activity (Nakamura et al., 2011), emotion (Vandewalle et al., 2010; Legates et al., 2012), and cognitive functions such as memory (Ruby et al., 2008; Legates et al., 2012; Fernandez et al., 2014).

Human neuroimaging studies have revealed diurnal variation at both regional and whole-brain levels (Muto et al., 2016; Carlucci et al., 2023; Rivera-Rivera et al., 2024). Cortical responses during task performance in functional MRI showed significant circadian rhythmicity, with different phases depending on the brain regions (Muto et al., 2016). The variance of resting-state functional MRI signals drops endogenously, coinciding with dawn and dusk, notably in sensory cortices (Cordani et al., 2018). Transcranial magnetic stimulation studies showed that the excitability of the motor areas changes within the circadian cycle (Lang et al., 2011; Ly et al., 2016). However, the diurnal variation of basic metabolism in the human cerebrum remains elusive.

In this study, we investigated the diurnal variation of human baseline cerebral activity by using perfusion imaging to measure cerebral blood flow (CBF). We compared the baseline activity collected four times a day, every 6 h, with a within-participant design by utilizing the data from our previous study investigating the SCN activity (Oka et al., 2024). We explored cerebral regions where diurnal variations in brain activity were observed. Furthermore, to address differential temporal patterns of baseline activity in the cerebral regions showing diurnal variations, we investigated the functional connectivity between the cerebral regions and the SCN by utilizing the database of the Human Connectome Project (HCP).

## Materials and Methods

### Experimental designs

**Figure 1.**
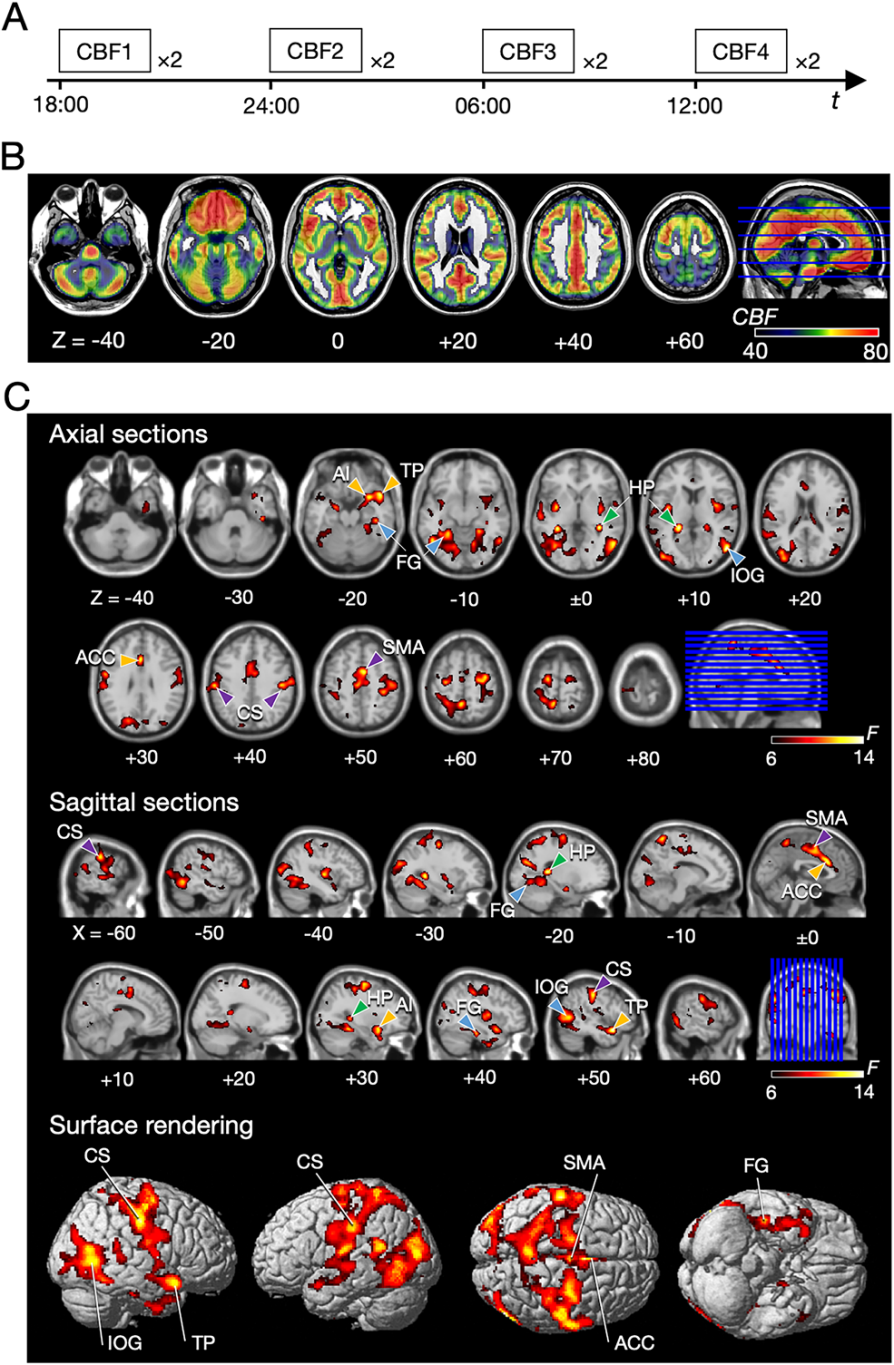
MRI scan schedule and results of CBF analysis. ***A***, Scan schedule. Scans were conducted in evening (18:00), midnight (24:00), morning (6:00), and daytime (12:00) sessions in that order. Two CBF images were acquired and averaged at each session to increase the signal-to-noise ratio. Between the scan sessions, the participants rested at the accommodation provided. ***B***, CBF image averaged across time points (unit: ml/100 mg/min). ***C***, *F*-maps of CBF in whole-brain ANOVA shown on axial and sagittal sections and surface rendering. The colors of arrowheads indicate the grouping of brain regions (green, the hippocampus; yellow, limbic areas; purple, somatomotor areas; cyan, visual areas).

This study examined the diurnal variation of human cerebral activity. We analyzed the published data from Oka et al. (2024). In the experiment, two perfusion images of pseudocontinuous arterial spin labeling (pCASL) were acquired for each participant four times within 24 h (18:00, 24:00, 6:00, 12:00 local time) to calculate the CBF at those specific time points (Fig. 1*A*). Perfusion imaging allows us to compare images taken at such long intervals. The lights in the MRI room were turned on during all scans. We instructed the participants to get sufficient sleep the night before the experiment. The participants were asked to maintain consistent calorie intake by eating meals similar to their usual diet, scheduled 4.5 h prior to each scan. They were instructed to rest overnight at a hotel between the 24:00 and 6:00 scans. To minimize fluctuations in the metabolic or physiological state, the participants were instructed not to engage in any vigorous physical activity throughout the protocol. Since the study required four meals across the day, those who preferred not to eat full meals late at night were allowed to consume light meals. The lighting conditions were kept constant inside the MRI environment, and the participants spent the interscan intervals under standard indoor lighting. No participant reported feeling unwell or emotionally distressed during the experiment.

### Participants

Twenty-seven right-handed participants without neurological or psychiatric disorders or sleep issues participated in the experiment (13 males and 14 females, aged 22.8 ± 2.7 years [mean ± standard deviation], ranging from 20 to 32 years). The participants were undergraduate or graduate students. None of the participants worked night shifts, which could have impacted their circadian rhythms. The Research Ethics Committee of the Faculty of Medicine at Juntendo University approved the experimental procedures. Written informed consent was obtained from all participants following the Declaration of Helsinki.

### MRI procedures

All MRI data were acquired using a 3 T MRI scanner at Juntendo University Hospital (Siemens Prisma) with a 32-channel head coil. T1-weighted structural images were obtained using 3D magnetization-prepared rapid gradient-echo (resolution, 0.8 × 0.8 × 0.8 mm^3^) in a separate scanning session. Whole-brain perfusion images were acquired using the pCASL technique with multiband imaging (number of measurements, 90; repetition time, 4.0 s; echo time, 25.2 ms; partial Fourier, 6/8; flip angle, 90°; labeling duration, 1.5 s; post labeling delay, 1.64 s; slice thickness, 1.82 mm; distance factor, 10%; number of slices, 72; slice acquisition order, ascending; in-plane field of view, 212 × 212 mm^2^; matrix size, 106 × 106; multiband factor, 6; Li et al., 2015). The imaging parameters for pCASL were determined based on the protocols of the Human Connectome Project (protocols.humanconnectome.org; Harms et al., 2018). Since the auto-align function of the MRI scanner was used to acquire the images, the head position at the start of image acquisition was almost the same at each time point. Two M0 images, included in the pCASL sequence, were acquired after the label/control image series. The mean image of these two M0 images was used for CBF quantification. Two images, one with anterior-to-posterior and the other with posterior-to-anterior encoding direction, were acquired using the spin-echo field map sequence before pCASL scans, taking <1 min. These images were used to perform the top-up distortion correction for pCASL images (Andersson et al., 2003). Each scan session took <20 min.

### Analyses of CBF data

The perfusion images were corrected for motion and distortion. The CBF images in the standard MNI (Montreal Neurological Institute) space were calculated using a command line interface of oxford_asl, which is part of the BASIL (Bayesian Inference for Arterial Spin Labeling) toolbox (Chappell et al., 2023) included in the FSL (Oxford Centre for the Functional Magnetic Resonance Imaging of the Brain Software Library; Smith et al., 2004). The absolute CBF values (ml/100 mg/min) were calculated in each cerebral voxel. To check for potential biases during spatial normalization, we computed the translation parameters required to coregister each perfusion image to the structure image at each time point. We then defined the subvoxel deviation as the remainder of the translation value divided by the voxel size (i.e., 2 mm). Subvoxel deviations did not differ significantly across time points on any axis (one-way repeated-measures analysis of variance (ANOVA); *x*-axis, *F*_(3,78)_ = 0.67, *p* = 0.57; *y*-axis, *F*_(3,78)_ = 0.97, *p* = 0.41; *z*-axis, *F*_(3,78)_ = 0.56, *p* = 0.64; Fig. S1), indicating that the observed time effects were not driven by any biases during spatial normalization. The structural image was used as a reference in the registration and generation of a cerebrospinal fluid mask for absolute quantification. Spatial smoothing [full-width at half-maximum of Gaussian kernel (FWHM) = 8.0 mm] was applied to the CBF images. For analyzing the SCN, minimal spatial smoothing (FWHM = 2.0 mm) was used to accurately localize signals within this structure. We performed a one-way repeated-measures ANOVA for the CBF activity in each cerebral voxel across the four time points (6:00, 12:00, 18:00, and 24:00 on local time) using Statistical Parametric Mapping 12 (SPM12; https://www.fil.ion.ucl.ac.uk/spm/). We used voxel-level and cluster-level thresholds to investigate significant CBF activity (*p* < 0.001 for cluster identification uncorrected for multiple comparisons and *p* < 0.05 for cluster-level significance with family-wise error correction). Contrast estimates, i.e., beta values with the mean adjusted to zero, were calculated for ANOVA in SPM12. Nonsphericity in the factorial design of ANOVA was corrected in SPM12. We investigated the temporal patterns of CBF activity using post hoc *t* tests among time points.

For the region of interest (ROI) analysis, the coordinates of the voxel for the SCN ROI at a 2 mm resolution for perfusion imaging were *x* = −2, *y *= + 2, *z* = −16 for the left ROI, and *x* = + 2, *y* = + 2, *z* = −16 for the right ROI. These coordinates were based on the hypothalamus parcellation results reported by Oka et al. (2024). The location of the SCN at 1.25 mm resolution was identified using a boundary mapping technique applied to the resting-state functional MRI data collected in our previous study (Ogawa et al., 2022). The protocol yielded the central coordinates of the SCN ROI at 1.25 mm resolution (left, *x* = −1.8, *y* = + 1.5, *z* = −15.8; right, *x* = +1.8, *y* = +1.5, *z* = −15.8). These coordinates were then transformed into the 2 mm resolution space. The CBF values of the left and right SCN were averaged. To compare the CBF activities between 12:00 and 24:00, we performed a paired *t* test in each cerebral voxel.

### Human Connectome Project data

Functional images were downloaded from the HCP 7T database. The resting-state data of 164 participants (61 males and 103 females) in various age groups (22−25 years: 18 participants; 26–30 years: 79; 31–35 years: 65; and ≥ 36 years: 2) were analyzed. Functional images were scanned using gradient-echo echoplanar imaging [repetition time (TR), 1,000 ms; echo time (TE), 22.2 ms; flip angle, 45°; field of view, 208 mm × 208 mm; voxel size, 1.6 mm isotropic; 85 slices; multiband factor (Moeller et al., 2010), 5; image acceleration factor, 2; partial Fourier sampling, 7/8; echo spacing, 0.64 ms; bandwidth, 1,924 Hz/Px].

### Analyses of HCP data

The images were preprocessed in the following steps: First, images were corrected for head motion and susceptibility-induced distortions and then spatially normalized into the standard MNI space. Next, the data were projected onto the standard surface (32,492 vertices in each hemisphere; Van Essen, 2005; Glasser et al., 2013). A temporal high-pass filter (cutoff frequency, 0.0005 Hz) was applied for the resting-state data to remove the linear trend. FMRIB’s ICA-based Xnoiseifier (FIX; Salimi-Khorshidi et al., 2014) was used to automatically reduce noise and nuisance components, such as head motion. Multimodal surface matching (MSM) was applied to adjust the projection on the standard surface for each participant (Robinson et al., 2014, 2018). Further preprocessing details are described previously (Glasser et al., 2013; Smith et al., 2013). The preprocessed files are available from the HCP web page (db.humanconnectome.org). The global signal was regressed out from the whole brain, and spatial smoothing (FWHM = 6.0 mm) was applied across the vertices in the cerebrum. The time series of fMRI signals of the SCN were extracted from volumetric images, and Pearson’s correlation coefficients with the time series of cerebral fMRI signals were calculated in a vertex-wise manner. The correlation coefficients were transformed into Fisher *z* values. The Connectome Workbench (Marcus et al., 2011) was used to visualize the functional connectivity (i.e., Fisher *z*-transformed correlation). Although the hypothalamus is a small brain structure, prior studies have successfully calculated the functional connectivity between its subdivisions and the cerebral cortex (Kullmann et al., 2014; Hirose et al., 2016; Zhang et al., 2018). Our previous studies have enabled us to calculate the functional connectivity of hypothalamic nuclei by localizing nuclei in the hypothalamus, including the SCN, using the resting-state functional MRI and parcellation methods (Osada et al., 2017, 2021, 2024; Ogawa et al., 2020, 2022; Suda et al., 2020; Nakajima et al., 2022; Rison et al., 2024; Asano et al., 2025). For the functional connectivity analyses, the centroid coordinates of the SCN at a 1.25 mm resolution (left: *x* = −1.8, *y *= +1.5, *z* = −15.8; right: *x *= +1.8, *y *= +1.5, *z* = −15.8) were converted to a 1.6 mm resolution to determine the voxels of the SCN ROI. The MRI signals from the left and right SCN were averaged to compute the functional connectivity.

## Results

This study investigated the diurnal variation of human CBF activity at rest. Figure 1*B* shows the averaged CBF across all four time points (see also Fig. S2 for CBF maps of all four time points). To identify brain regions showing diurnal variation, we performed a one-way ANOVA on the data from the four time points. The result showed four significant clusters with eleven noticeable peaks (Fig. 1*C*, Table 1).

**Table 1.** Significant clusters and their peaks

	MNI coordinates of peak			Peak *Z* value	Cluster	
	*x*	*y*	*z*		size	P-FWE
Cluster 1 Fusiform gyrus	44	−24	−26	5.09	4,184	<0.001
Inferior occipital gyrus	54	−70	8	5.06		
Hippocampus	34	−38	0	4.93		
Cluster 2 Temporal pole	50	14	−18	5.06	5,879	<0.001
Central sulcus	46	−20	44	4.87		
Anterior insula	32	10	−18	4.76		
Cluster 3 Hippocampus	−22	−38	8	5.04	6,679	<0.001
Fusiform gyrus	−24	−50	−14	4.82		
Cluster 4 Supplementary motor area	6	0	54	4.96	8,313	<0.001
Central sulcus	−58	−22	36	4.94		
Anterior cingulate cortex	0	20	30	4.76		

MNI, Montreal Neurological Institute; FWE, family-wise error.

**Figure 2.**
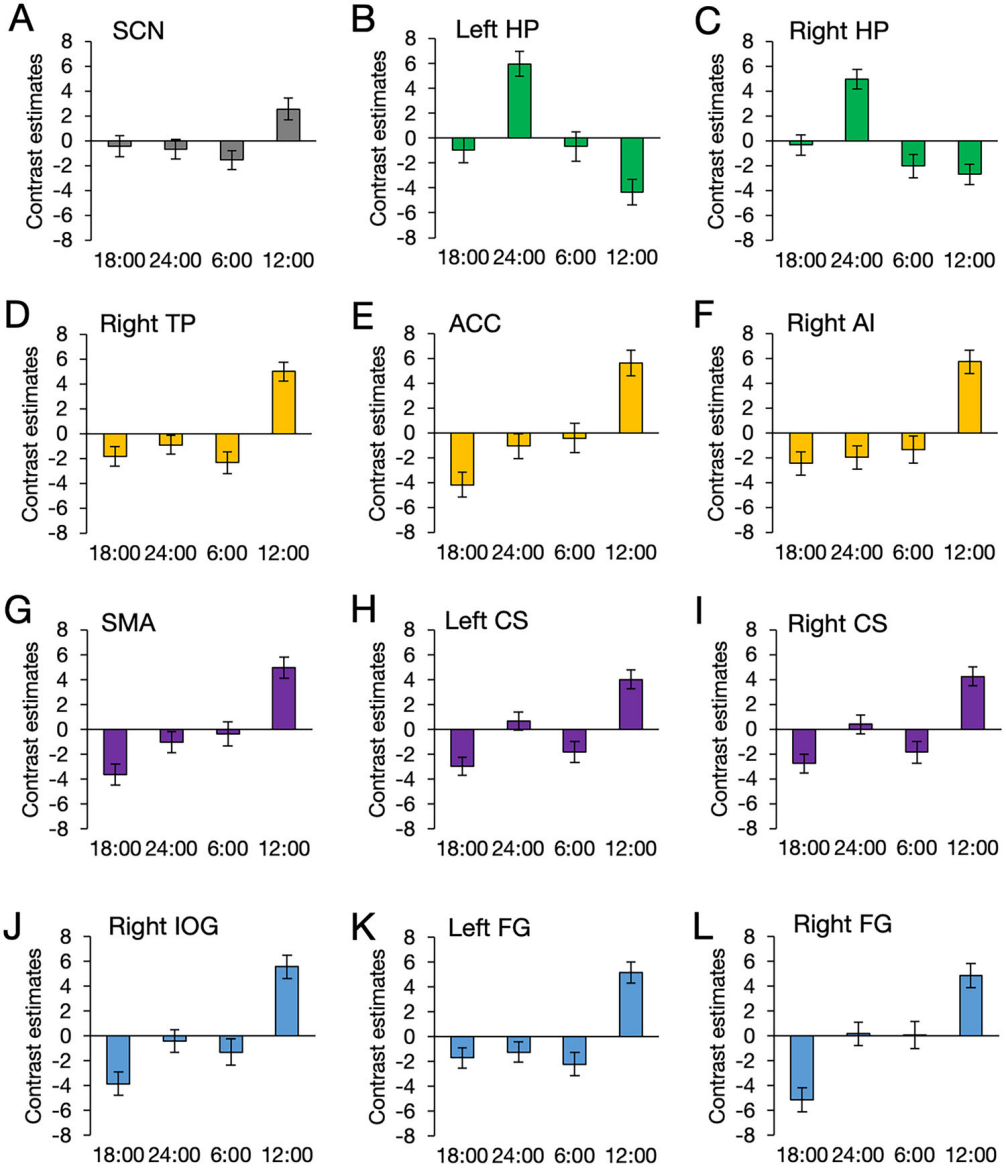
ROI analyses for significant clusters of whole brain result of CBF. ***A***, Contrast estimates of the SCN (left and right averaged). The contrast estimates at 12:00 and 6:00 were the highest and lowest, respectively. ***B***, ***C***, Contrast estimates of the hippocampus. In contrast to the SCN, the hippocampus showed the highest and lowest contrast estimates at 24:00 and 12:00, respectively. ***D–L***, Contrast estimates of the other areas found in the CBF analysis. Similar to the SCN, the contrast estimate at 12:00 was the highest. Error bars indicate standard errors of the mean. ACC, anterior cingulate cortex; AI, anterior insular; CS, central sulcus; FG, fusiform gyrus; TP, temporal pole; HP, hippocampus; IOG, inferior occipital gyrus; SMA, supplementary motor area. Note that these plots illustrate the temporal patterns and peak timing of CBF activity within each cluster. The effect sizes shown here should not be interpreted as unbiased estimates of the magnitude of the diurnal effects.

Our previous study found the diurnal variation of the human SCN (Fig. 2*A*; Oka et al., 2024). The present study found diurnal variation in the limbic (cingulate, insular, and temporopolar) and sensorimotor (visual and somatosensory/motor) areas. The bilateral hippocampi showed the invert phase, highest at 24:00 (Fig. 2*B*,*C*), while diurnal variation similar to the SCN, highest at 12:00, was observed in the right temporal pole (Fig. 2*D*), anterior cingulate cortex (Fig. 2*E*), right anterior insula (Fig. 2*F*), supplementary motor area (Fig. 2*G*), left central sulcus region (Fig. 2*H*), right central sulcus region (Fig. 2*I*), right inferior occipital gyrus (Fig. 2*J*), left fusiform gyrus (Fig. 2*K*), and right fusiform gyrus (Fig. 2*L*). In contrast, diurnal variation was not evident in most of the association areas in the cerebral cortex. Post hoc comparisons among the four time points are conducted at the cerebral areas and summarized in Table S1*A*.

**Figure 3.**
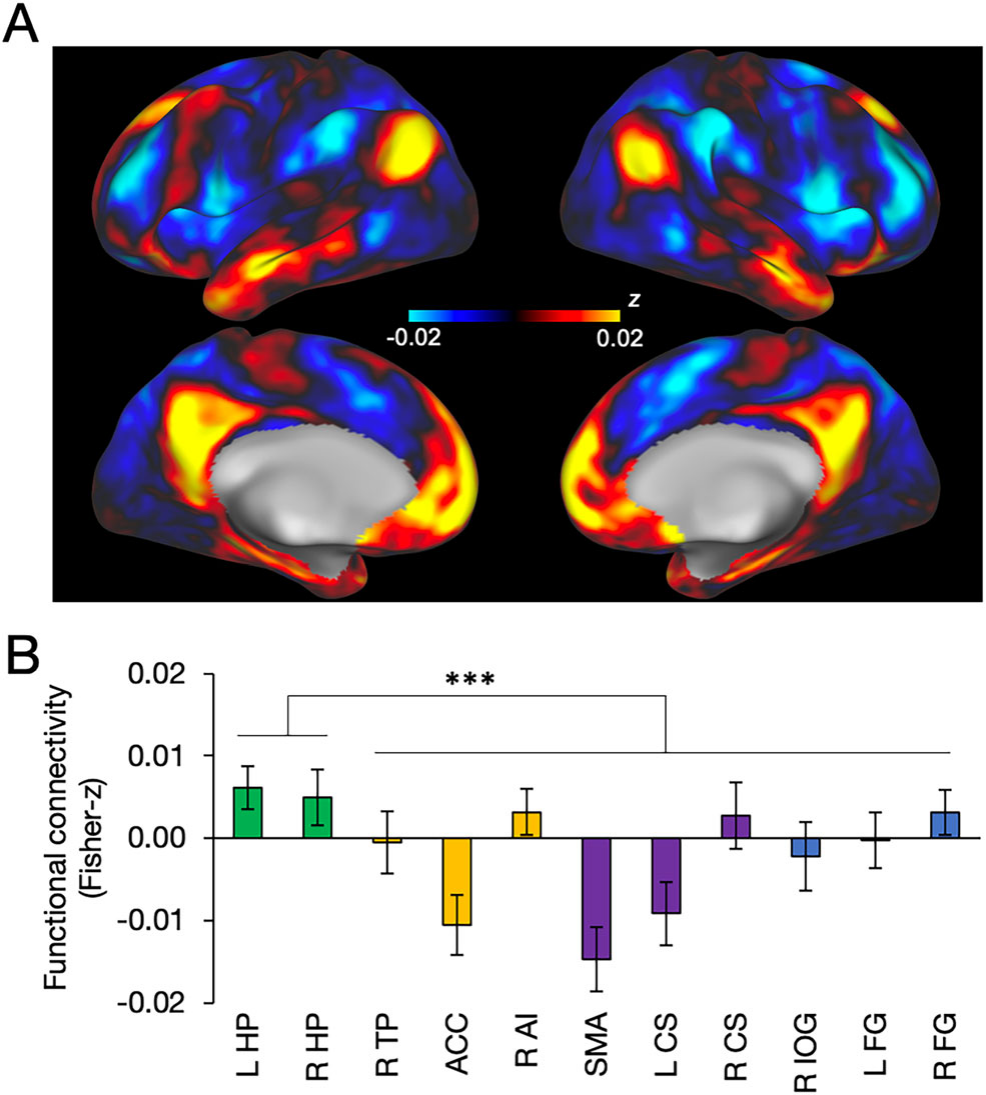
Resting-state functional connectivity between the cerebrum and SCN. ***A***, Functional connectivity shown on the cerebral surfaces. ***B***, Regional functional connectivity. The hippocampus showed significantly greater functional connectivity than the other regions. Error bars indicate standard errors of the mean. Asterisks indicate statistical significance (***p < 0.001).

Functional connectivity, where the SCN (averaged across both left and right) was used as a seed, was calculated throughout the whole brain as a Fisher *z*-transformed temporal correlation using the HCP dataset. Higher functional connectivity was observed mainly in the regions of default-mode network (e.g., the medial prefrontal cortex and precuneus; Fig. 3*A*). Among the regions that showed the diurnal variation of brain activity, the hippocampus showed significantly greater functional connectivity with the SCN than the other regions (*t*_(163)_ = 3.9, *p* = 0.00015; Fig. 3*B*). To examine whether functional connectivity itself shows diurnal variation, we separately analyzed the functional connectivity of cerebral regions with the SCN according to the time points in the CBF experiments. The functional connectivity between 9:00 and 15:00, which corresponds to the CBF measurement at 12:00 (Fig. S3*A*), was similar to the functional connectivity between 15:00 and 21:00, which corresponds to the CBF measurement at 18:00 (Fig. S3*B*), as shown in a difference map (Fig. S3*C*). Few statistically significant differences were observed (Fig. S3*D*). In relation to this analysis, we examined the effect of the global signal of the brain on the functional connectivity and observed little influence (Fig. S3*E–H*).

To confirm that the observed functional connectivity patterns were specific to the SCN and not driven by physiological artifacts (e.g., pulsation, respiration), partial volume effects, or generic hypothalamic signals, we performed two control analyses. First, to assess the potential influence of cerebrospinal fluid (CSF) signals, we analyzed functional connectivity using a seed displaced into the CSF (shifted two voxels ventrally and four voxels laterally from the SCN; Fig. S3*I*, left). The resulting connectivity map showed negligible spatial correlation with the SCN connectivity map (*r* = 0.044; Fig. S3*J*), indicating that the SCN results were not attributable to diffuse physiological noise or CSF contamination. Second, to determine whether the findings reflected SCN-specific rather than general hypothalamic connectivity, we examined the functional connectivity using a seed in the posterior hypothalamus (PH; Fig. S3*I*, right). Although a moderate spatial correlation was observed (*r* = 0.51), the connectivity profile of the PH was distinct from that of the SCN (Fig. S3*K*). Together, these results support the spatial specificity of the SCN functional connectivity.

To assess the internal consistency of the SCN functional connectivity, we quantified the spatial similarity of whole-brain functional connectivity maps across participants using pairwise Pearson’s correlations. As a benchmark, we performed the same analysis using the nucleus accumbens (NAc) as a seed with an equivalent number of voxels. The correlation values were transformed into Fisher *z*. The SCN maps showed significant reproducibility across participants (average Fisher *z* = 0.049, one-sample *t* test, *t*_(13365)_ = 31.9, *p* < 0.0001; Fig. S3*L*). Although this consistency was lower than that observed for the NAc (average Fisher *z* = 0.126, one-sample *t* test, *t*_(13365)_ = 115.0, *p* < 0.0001), likely reflecting the lower signal-to-noise ratio of this small hypothalamic nucleus, the results confirm that the SCN connectivity pattern was robustly conserved across individuals (Fig. S3*L,M*).

**Figure 4.**
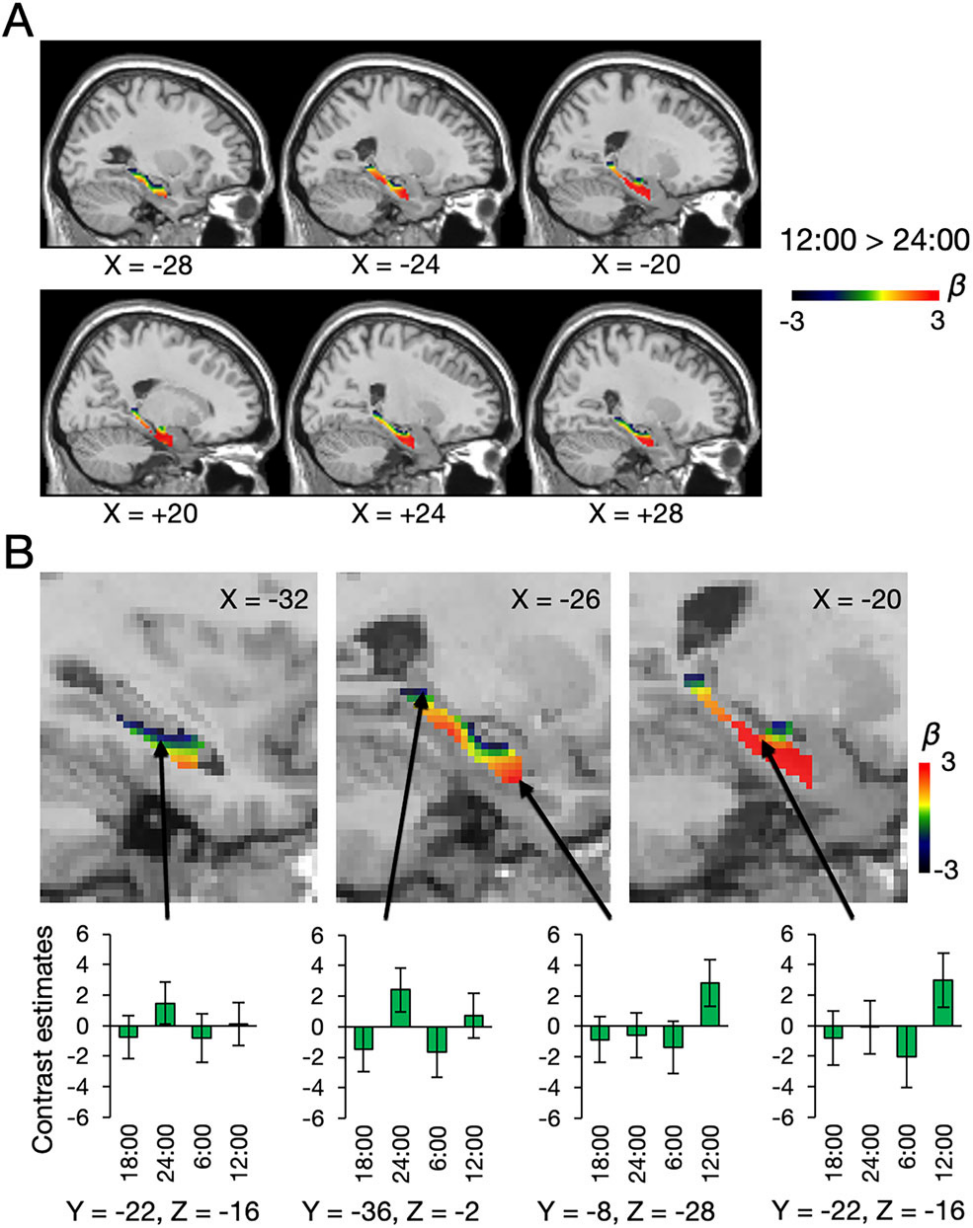
Beta map of the contrast 12:00 > 24:00 in the hippocampus. ***A***, Results of left and right hippocampi shown on sagittal sections. ***B***, Enlarged beta map of the contrast. The anterior hippocampus shows more positive contrast estimates than the dorsal and posterior parts. Error bars indicate standard errors of the mean.

We further investigated the diurnal variation of the CBF activity within the hippocampus in more detail. We contrasted the activity between 12:00 and 24:00 to visualize the spatial pattern of the diurnal variation in the hippocampal region as defined in the HCP parcellation (Glasser et al., 2016a,b; Fig. 4*A*). The results showed that the activity in the anterior and medial hippocampus tended to be highest at 12:00 (Fig. 4*B*). In contrast, the activity in the posterior and lateral hippocampus tended to peak at 24:00. The main effects were calculated at the demonstrated four coordinates in the hippocampus (*x* = −32, *y* = −22, *z* = −16, *F*_(3,78)_ = 1.21, *p* = 0.31; *x* = −26, *y* = −36, *z* = −2, *F*_(3,78)_ = 3.66, *p* = 0.0159; *x* = −26, *y* = −8, *z* = −28, *F*_(3,78)_ = 3.21, *p* = 0.0275, *x* = −20, *y* = −22, *z* = −16, *F*_(3,78)_ = 2.77, *p* = 0.0471). Post hoc comparisons among the four time points are reported in Table S1*B*. Figure S4, *A–C*, shows reproducible beta maps of the contrast 12:00 > 24:00 using only the first or second image in each session.

**Figure 5.**
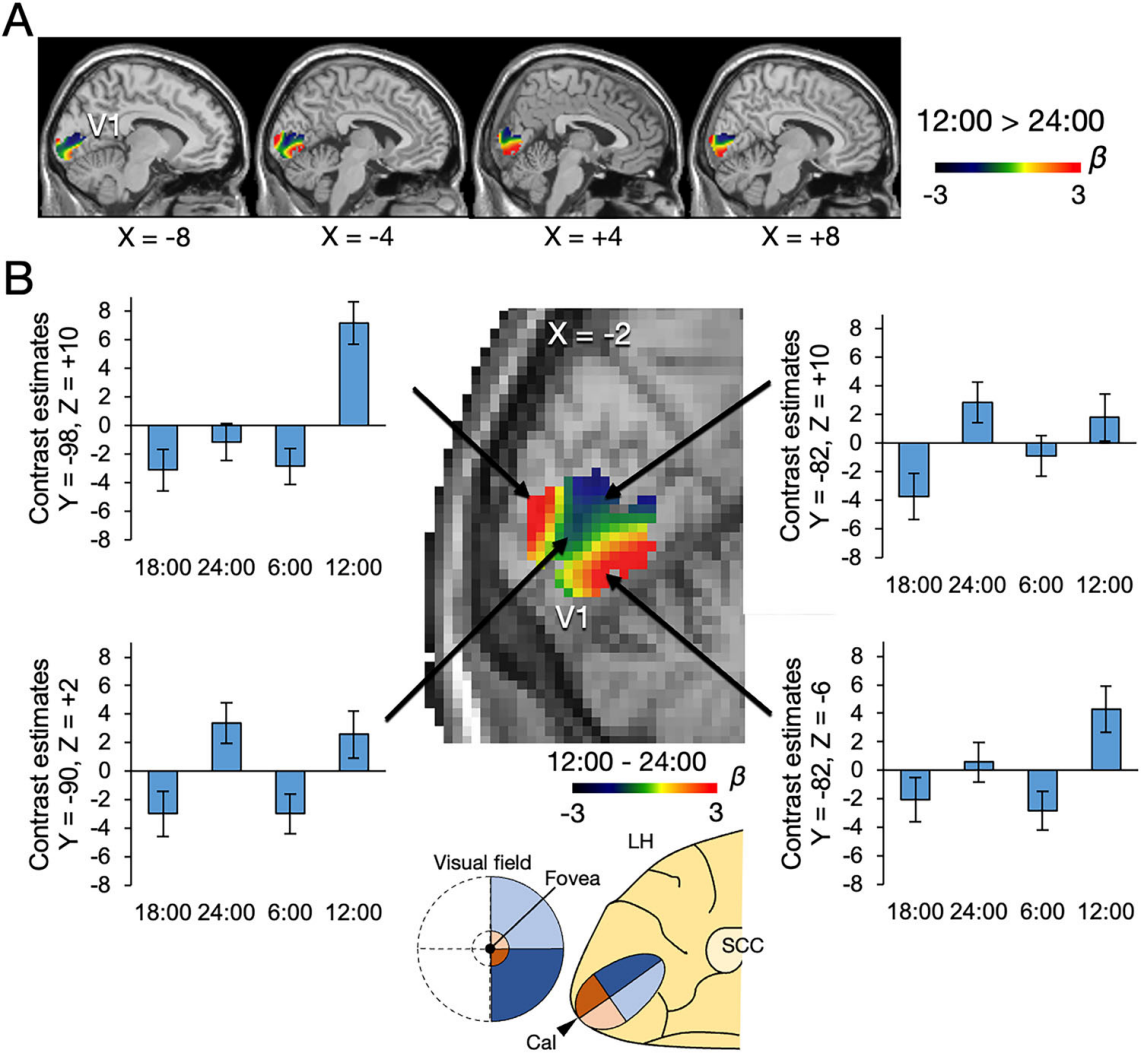
Beta map of the contrast 12:00 > 24:00 in the primary visual area (V1). ***A***, Results of left and right V1 shown on sagittal sections. ***B***, Enlarged beta map of the contrast. The patterns of the contrast estimates were spatially different in the V1. The activity gradient seems to be associated with the vertical and horizontal meridian in the visual field. Error bars indicate standard errors of the mean.

In the whole-brain analysis contrasting 12:00 and 24:00, we observed a distinctive and spatial localized pattern of mixed positive and negative effects in the primary visual cortex (V1), along with the hippocampus (Fig. S4*D*). To further characterize this unique signal property, we investigated the diurnal variation within the V1 as defined in Rosenke et al. (2021). We contrasted the activity between 12:00 and 24:00 to visualize the spatial pattern of the diurnal variation in the V1 (Fig. 5*A*). The patterns of the contrast estimates showed different patterns depending on the locations in the visual field. The subregions associated with the vertical meridian showed higher activity at 12:00, whereas the activity of the subregions associated with the horizontal meridian peaked at 24:00 (Fig. 5*B*). The main effects were calculated at the demonstrated four coordinates in the V1 (*x* = −2, *y* = −98, *z* = + 10, *F*_(3,78)_ = 10.4, *p* = 8.08 × 10−6; *x* = −2, *y* = −90, *z *= +2, *F*_(3,78)_ = 4.68, *p* = 0.0047; *x* = −2, *y* = −82, *z *= +10, *F*_(3,78)_ = 3.37, *p* = 0.0226; *x* = −2, *y* = −82, *z* = −6, *F*_(3,78)_ = 3.65, *p* = 0.0161). Post hoc comparisons among the four time points are reported in Table S1*C*. Figure S4, *E–G*, shows the reproducibility of the beta maps of the contrast 12:00 > 24:00 using only the first or second image in each session. In the surrounding early visual areas (V2 and V3), the CBF activity was higher at 12:00 than at 24:00, with more spatially uniform patterns (Fig. S4*H*). The spatial and temporal gradient of the activity seems noticeable in the V1. In contrast, the regions shown in Figure 2, exhibiting the diurnal variation like the SCN, tended to show more spatially homogeneous temporal contrasts (Fig. S4*I–Q*).

To investigate the differences in diurnal variations in CBF across the entire brain, we categorized the cerebrocortical areas into three groups (older, sensorimotor, and higher-order areas; Fig. S5). For each group, we quantified the number of voxels showing significant diurnal variations in CBF (Fig. 1*C*). The proportions of significant voxels in the three groups were 18.3, 51.3, and 30.3%, while the proportions of total cortical voxels belonging to these three groups were 26.7, 36.1, and 37.3%, respectively (Fig. S5). When comparing higher-level areas to older and sensorimotor areas, the spatial distribution of voxels exhibiting diurnal variation differed significantly across three groups and could not be explained simply by regional size (chi-squared test, χ^2^_(1)_ = 5.8 × 10^2^, *p* << 0.05).

## Discussion

This study investigated the diurnal variation of the human CBF activity at rest. We observed a temporal activity pattern in the limbic and sensorimotor areas, with higher activity at noon and lower activity in the early morning, as seen in the SCN. The hippocampus showed higher activity at midnight and lower activity at noon, as opposed to the SCN. Analyses of the resting-state functional connectivity between the SCN and the cerebral regions using the database of the HCP showed greater functional connectivity in the hippocampus than in the other regions. These results indicate that older cerebral regions exhibit distinct patterns of diurnal variation depending on the SCN activity and their functional connectivity with the SCN.

It has been shown that the human SCN shows brain activity higher at noon and lower at midnight/dawn (Hofman and Swaab, 1993, 1994; Hofman, 2000; Oka et al., 2024), similar to the animal SCN. In the present study, we observed that the hippocampus, particularly its posterior part, showed higher activity at midnight and lower activity at noon, consistent with animal studies showing that the hippocampal activity during the night was greater than during daylight time (Barnes et al., 1977; Endo et al., 1990; Chaudhury et al., 2005; Naseri Kouzehgarani et al., 2020). We also observed that the diurnal variation in the human cortical activity was similar to that in the SCN. Although animal studies examining cortical brain activity at rest are rare, our results suggest the possible relationship between circadian rhythms and memory formation in the hippocampus (Gerstner et al., 2009; Gerstner and Yin, 2010).

In line with our findings that the temporal pattern of diurnal variation differed within the hippocampus, previous studies have indicated functional differences along the long axis of the hippocampus. One review identified the global representation of the environment in the anterior hippocampus and the fine-grained local representations of the environment in the posterior hippocampus (Poppenk et al., 2013). Another review found that the anterior hippocampus is more strongly connected with a cerebral network linked to remembering, while the posterior hippocampus is associated with a distinct network related to behavioral salience (Angeli et al., 2025). The differential diurnal variations in the hippocampal CBF activity shown in the present study may be linked to this functional heterogeneity within the hippocampus.

Several studies have explored diurnal variations in functional connectivity, CBF, and regional fMRI signal amplitude (Shannon et al., 2013; Hodkinson et al., 2014; Jiang et al., 2016; Orban et al., 2020). For example, default mode regions changed their functional connectivity and CBF from morning to afternoon (Hodkinson et al., 2014), and the medial temporal regions exhibited stronger correlations with the frontal and parietal brain regions in the evening than in the morning (Shannon et al., 2013). Decreases in resting-state functional connectivity across the whole brain have been observed from the morning to evening (Orban et al., 2020). In addition, regional fMRI signal amplitudes in the visual and somatomotor areas were higher in the morning than in the evening (Jiang et al., 2016). In this study, measuring CBF activity four times a day, every 6 h, in the same participants enabled us to characterize diurnal activity patterns with greater temporal resolution. We observed that the visual, limbic, and sensorimotor areas showed higher activity at noon, and the hippocampus showed higher activity at midnight than at other times during the day. These findings align with and extend previous reports on diurnally varying regional neural activity.

Circadian rhythms influence a broad range of brain functions. Transcranial magnetic stimulation studies showed that the excitability in the motor cortex varies within the circadian cycle (Lang et al., 2011; Ly et al., 2016). Functional MRI studies reported the circadian modulation of limbic reward-related brain activity (Byrne et al., 2019; Puig et al., 2023). A magnetic resonance spectroscopy study showed a significant difference in metabolite ratios in the parietal lobule between morning and evening (Arm et al., 2019). These may reflect the diurnal variation of the baseline activity in the cerebrocortical regions observed in the present study. It should be noted that the core body temperature, end-tidal carbon dioxide, respiratory dynamics, heart-rate variability, arousal, and sleepiness were not acquired in the MRI scanner. As these systemic physiological factors can exhibit strong circadian modulation, they may influence regional CBF and functional connectivity independent of local neural activity. A previous study has demonstrated that circadian and homeostatic processes interact within the SCN (Schmidt et al., 2009). While our design aimed to minimize homeostatic influences, including standardized sleep instructions and controlled overnight rest, residual interactions between circadian and homeostatic processes may still have contributed to the observed CBF variations.

Functional connectivity with the SCN is present in many association areas in the cerebral cortex, even though diurnal variation was not evident in these areas. Specifically, the SCN was functionally connected with the default-mode and limbic network regions (Yeo et al., 2011; Horn et al., 2014), including the medial prefrontal cortex, precuneus, and hippocampus (Ezama et al., 2021; Fig. 3*A*). The connectivity pattern is largely consistent with our previous study showing higher functional connectivity between the other hypothalamic nuclei and the default-mode/limbic network regions (Asano et al., 2023). Of the cerebral regions that did show diurnal variation in the CBF activity, functional connectivity between the SCN and the hippocampus was stronger than that with the other cerebral regions in the limbic and sensorimotor areas. The SCN exerts both phase-leading and phase-lagging influences on downstream networks, depending on the target region and its function. Such differential phase relationships could lead to antiphase patterns in CBF activity, particularly when measured at discrete time points. One plausible neurobiological mechanism involves the predominantly GABAergic nature of SCN projections (Moore and Speh, 1993). Although most SCN neurons release GABA, their downstream effects can be inhibitory or excitatory, depending on the receptor environment and time of day. Therefore, these dynamic interactions may lead to region-specific CBF responses.

An increase in CBF is a primary physiological driver of a positive fMRI (i.e., blood oxygenation level-dependent, BOLD) response, whereas additional factors, such as the cerebral metabolic rate of oxygen consumption and cerebral blood volume, can also influence the BOLD signal change. In the present study, CBF activity and functional connectivity were derived from two independent datasets; thus, the interpretation reflects converging evidence across modalities rather than a within-participant linkage between CBF and BOLD measures. The direct relationship between the CBF activity and functional connectivity may underlie the observed phase differences in the diurnal variation, with greater connectivity potentially contributing to the antiphase pattern of the diurnal variation. Although further studies are required at the cellular and molecular levels to clarify the specific mechanisms involved, the phylogenetically older areas may be selectively responsive to the interaction from the SCN.

## Data Availability

The data and code that support the findings in this study are available at Dryad (DOI: 10.5061/dryad.hx3ffbgtn), excluding raw image data. The raw image data cannot be deposited in a public repository because sharing it was not included in the informed consent. Any additional information needed to examine the results reported in this paper can be requested to the corresponding author upon reasonable request. The HCP data can be found at balsa.wustl.edu.

## References

[B1] Andersson JLR, Skare S, Ashburner J (2003) How to correct susceptibility distortions in spin-echo echo-planar images: application to diffusion tensor imaging. Neuroimage 20:870–888. 10.1016/S1053-8119(03)00336-7 14568458

[B2] Angeli PA, DiNicola LM, Saadon-Grosman N, Eldaief MC, Buckner RL (2025) Specialization of the human hippocampal long axis revisited. Proc Natl Acad Sci U S A 122:e2422083122. 10.1073/pnas.2422083122 39808662 PMC11760929

[B3] Arm J, Al-iedani O, Lea R, Lechner-Scott J, Ramadan S (2019) Diurnal variability of cerebral metabolites in healthy human brain with 2D localized correlation spectroscopy (2D L-COSY). J Magn Reson Imaging 50:592–601. 10.1002/jmri.26642 30629765

[B4] Asano S, et al. (2023) Reduced gray matter volume in the default-mode network associated with insulin resistance. Cereb Cortex 33:11225–11234. 10.1093/cercor/bhad358 37757477

[B5] Asano S, et al. (2025) Insulin resistance-related gray matter volume reduction is associated with the default mode network. Juntendo Med J 71:JMJ24-0044-OT. 10.14789/ejmj.JMJ24-0044-OT PMC11915748 40109401

[B6] Barnes CA, McNaughton BL, Goddard G V, Douglas RM, Adamec R (1977) Circadian rhythm of synaptic excitability in rat and monkey central nervous system. Science 197:91–92. 10.1126/science.194313 194313

[B7] Bering T, Carstensen MB, Wörtwein G, Weikop P, Rath MF (2017) The circadian oscillator of the cerebral cortex: molecular, biochemical and behavioral effects of deleting the arntl clock gene in cortical neurons. Cereb Cortex 28:644–657. 10.1093/cercor/bhw406 28052921

[B8] Bering T, Gadgaard C, Vorum H, Honoré B, Rath MF (2023) Diurnal proteome profile of the mouse cerebral cortex: conditional deletion of the Bmal1 circadian clock gene elevates astrocyte protein levels and cell abundance in the neocortex and hippocampus. Glia 71:2623–2641. 10.1002/glia.24443 37470358

[B9] Byrne JEM, Tremain H, Leitan ND, Keating C, Johnson SL, Murray G (2019) Circadian modulation of human reward function: is there an evidentiary signal in existing neuroimaging studies? Neurosci Biobehav Rev 99:251–274. 10.1016/j.neubiorev.2019.01.025 30721729

[B10] Cai X, Qiao J, Kulkarni P, Harding IC, Ebong E, Ferris CF (2020) Imaging the effect of the circadian light-dark cycle on the glymphatic system in awake rats. Proc Natl Acad Sci U S A 117:668–676. 10.1073/pnas.1914017117 31848247 PMC6955326

[B11] Campbell I, et al. (2024) Regional response to light illuminance across the human hypothalamus. Elife 13:RP96576. 10.7554/eLife.96576.3 39466317 PMC11517251

[B12] Carlucci M, Lett T, Chavez S, Malinowski A, Lobaugh NJ, Petronis A (2023) Diurnal oscillations of MRI metrics in the brains of male participants. Nat Commun 14:7044. 10.1038/s41467-023-42588-6 37923728 PMC10624685

[B13] Cermakian N, Waddington Lamont E, Boudreau P, Boivin DB (2011) Circadian clock gene expression in brain regions of Alzheimer’s disease patients and control subjects. J Biol Rhythms 26:160–170. 10.1177/0748730410395732 21454296

[B14] Chappell MA, Kirk TF, Craig MS, McConnell FAK, Zhao MY, MacIntosh BJ, Okell TW, Woolrich MW (2023) BASIL: a toolbox for perfusion quantification using arterial spin labelling. Imag Neurosci 1:41. 10.1162/imag_a_00041 PMC12007520 40799705

[B15] Chaudhury D, Wang LM, Colwell CS (2005) Circadian regulation of hippocampal long-term potentiation. J Biol Rhythms 20:225–236. 10.1177/0748730405276352 15851529 PMC2581477

[B16] Chen C-Y, Logan RW, Ma T, Lewis DA, Tseng GC, Sibille E, McClung CA (2016) Effects of aging on circadian patterns of gene expression in the human prefrontal cortex. Proc Natl Acad Sci U S A 113:206–211. 10.1073/pnas.1508249112 26699485 PMC4711850

[B17] Cordani L, Tagliazucchi E, Vetter C, Hassemer C, Roenneberg T, Stehle JH, Kell CA (2018) Endogenous modulation of human visual cortex activity improves perception at twilight. Nat Commun 9:1274. 10.1038/s41467-018-03660-8 29636448 PMC5893589

[B18] Dewandre D, Atienza M, Sanchez-Espinosa MP, Cantero JL (2018) Effects of PER3 clock gene polymorphisms on aging-related changes of the cerebral cortex. Brain Struct Funct 223:597–607. 10.1007/s00429-017-1513-0 28900721

[B19] Endo Y, Jinnai K, Endo M, Fujita K, Kimura F (1990) Diurnal variation of cerebral blood flow in rat hippocampus. Stroke 21:1464–1469. 10.1161/01.STR.21.10.1464 2219212

[B20] Enoki R, Kuroda S, Ono D, Hasan MT, Ueda T, Honma S, Honma KI (2012) Topological specificity and hierarchical network of the circadian calcium rhythm in the suprachiasmatic nucleus. Proc Natl Acad Sci U S A 109:21498–21503. 10.1073/pnas.1214415110 23213253 PMC3535646

[B21] Ezama L, Hernández-Cabrera JA, Seoane S, Pereda E, Janssen N (2021) Functional connectivity of the hippocampus and its subfields in resting-state networks. Eur J Neurosci 53:3378–3393. 10.1111/ejn.15213 33786931 PMC8252772

[B22] Fernandez F, Lu D, Ha P, Costacurta P, Chavez R, Heller HC, Ruby NF (2014) Dysrhythmia in the suprachiasmatic nucleus inhibits memory processing. Science 346:854–857. 10.1126/science.1259652 25395537 PMC4459503

[B23] Fishbein AB, Knutson KL, Zee PC (2021) Circadian disruption and human health. J Clin Invest 131:e148286. 10.1172/JCI148286 34596053 PMC8483747

[B24] Franken P, Dijk D-J (2009) Circadian clock genes and sleep homeostasis. Eur J Neurosci 29:1820–1829. 10.1111/j.1460-9568.2009.06723.x 19473235

[B25] Germain A, Nofzinger EA, Meltzer CC, Wood A, Kupfer DJ, Moore RY, Buysse DJ (2007) Diurnal variation in regional brain glucose metabolism in depression. Biol Psychiatry 62:438–445. 10.1016/j.biopsych.2006.09.043 17217926 PMC3195370

[B27] Gerstner JR, Yin JCP (2010) Circadian rhythms and memory formation. Nat Rev Neurosci 11:577–588. 10.1038/nrn2881 20648063 PMC6544049

[B26] Gerstner JR, Lyons LC, Wright KP, Loh DH, Rawashdeh O, Eckel-Mahan KL, Roman GW (2009) Cycling behavior and memory formation: table 1. J Neurosci 29:12824–12830. 10.1523/JNEUROSCI.3353-09.2009 19828795 PMC4077269

[B28] Glasser MF, et al. (2013) The minimal preprocessing pipelines for the Human Connectome Project. Neuroimage 80:105–124. 10.1016/j.neuroimage.2013.04.127 23668970 PMC3720813

[B29] Glasser MF, et al. (2016a) A multi-modal parcellation of human cerebral cortex. Nature 536:171–178. 10.1038/nature18933 27437579 PMC4990127

[B30] Glasser MF, et al. (2016b) The Human Connectome Project’s neuroimaging approach. Nat Neurosci 19:1175–1187. 10.1038/nn.4361 27571196 PMC6172654

[B31] Harms MP, et al. (2018) Extending the Human Connectome Project across ages: imaging protocols for the lifespan development and aging projects. Neuroimage 183:972–984. 10.1016/j.neuroimage.2018.09.060 30261308 PMC6484842

[B32] Hastings MH, Maywood ES, Brancaccio M (2018) Generation of circadian rhythms in the suprachiasmatic nucleus. Nat Rev Neurosci 19:453–469. 10.1038/s41583-018-0026-z 29934559

[B33] Hirose S, et al. (2016) Lateral–medial dissociation in orbitofrontal cortex–hypothalamus connectivity. Front Hum Neurosci 10:244. 10.3389/fnhum.2016.00244 27303281 PMC4880561

[B34] Hodkinson DJ, O’Daly O, Zunszain PA, Pariante CM, Lazurenko V, Zelaya FO, Howard MA, Williams SCR (2014) Circadian and homeostatic modulation of functional connectivity and regional cerebral blood flow in humans under normal entrained conditions. J Cereb Blood Flow Metab 34:1493–1499. 10.1038/jcbfm.2014.109 24938404 PMC4158665

[B35] Hofman MA (2000) The human circadian clock and aging. Chronobiol Int 17:245–259. 10.1081/CBI-100101047 10841206

[B36] Hofman MA, Swaab DF (1993) Diurnal and seasonal rhythms of neuronal activity in the suprachiasmatic nucleus of humans. J Biol Rhythms 8:283–295. 10.1177/074873049300800402 8032088

[B37] Hofman MA, Swaab DF (1994) Alterations in circadian rhythmicity of the vasopressin-producing neurons of the human suprachiasmatic nucleus (SCN) with aging. Brain Res 651:134–142. 10.1016/0006-8993(94)90689-0 7922560

[B38] Horn A, Ostwald D, Reisert M, Blankenburg F (2014) The structural–functional connectome and the default mode network of the human brain. Neuroimage 102:142–151. 10.1016/j.neuroimage.2013.09.069 24099851

[B39] Inouye ST, Kawamura H (1979) Persistence of circadian rhythmicity in a mammalian hypothalamic “island” containing the suprachiasmatic nucleus. Proc Natl Acad Sci U S A 76:5962–5966. 10.1073/pnas.76.11.5962 293695 PMC411773

[B40] Inouye ST, Kawamura H (1982) Characteristics of a circadian pacemaker in the suprachiasmatic nucleus. J Comp Physiol A 146:153–160. 10.1007/BF00610233

[B41] Jiang C, Yi L, Su S, Shi C, Long X, Xie G, Zhang L (2016) Diurnal variations in neural activity of healthy human brain decoded with resting-state blood oxygen level dependent fMRI. Front Hum Neurosci 10:634. 10.3389/fnhum.2016.00634 28066207 PMC5169030

[B42] Kim JS, Han JW, Oh DJ, Suh SW, Kwon MJ, Park J, Jo S, Kim JH, Kim KW (2024) Effects of sleep quality on diurnal variation of brain volume in older adults: a retrospective cross-sectional study. Neuroimage 288:120533. 10.1016/j.neuroimage.2024.120533 38340880

[B43] Kullmann S, Heni M, Linder K, Zipfel S, Häring HU, Veit R, Fritsche A, Preissl H (2014) Resting-state functional connectivity of the human hypothalamus. Hum Brain Mapp 35:6088–6096. 10.1002/hbm.22607 25131690 PMC6869436

[B44] Lang N, Rothkegel H, Reiber H, Hasan A, Sueske E, Tergau F, Ehrenreich H, Wuttke W, Paulus W (2011) Circadian modulation of GABA-mediated cortical inhibition. Cereb Cortex 21:2299–2306. 10.1093/cercor/bhr003 21350047

[B45] Legates TA, Altimus CM, Wang H, Lee HK, Yang S, Zhao H, Kirkwood A, Weber ET, Hattar S (2012) Aberrant light directly impairs mood and learning through melanopsin-expressing neurons. Nature 491:594–598. 10.1038/nature11673 23151476 PMC3549331

[B46] Li JZ, et al. (2013) Circadian patterns of gene expression in the human brain and disruption in major depressive disorder. Proc Natl Acad Sci U S A 110:9950–9955. 10.1073/pnas.1305814110 23671070 PMC3683716

[B47] Li X, Wang D, Auerbach EJ, Moeller S, Ugurbil K, Metzger GJ (2015) Theoretical and experimental evaluation of multi-band EPI for high-resolution whole brain pCASL imaging. Neuroimage 106:170–181. 10.1016/j.neuroimage.2014.10.029 25462690 PMC4337884

[B48] Lim ASP, Myers AJ, Yu L, Buchman AS, Duffy JF, De Jager PL, Bennett DA (2013) Sex difference in daily rhythms of clock gene expression in the aged human cerebral cortex. J Biol Rhythms 28:117–129. 10.1177/0748730413478552 23606611 PMC3774838

[B49] Liu H, Liu J, Peng L, Feng Z, Cao L, Liu H, Shen H, Hu D, Zeng L-L, Wang W (2019) Changes in default mode network connectivity in different glucose metabolism status and diabetes duration. Neuroimage Clin 21:101629. 10.1016/j.nicl.2018.101629 30573410 PMC6411780

[B50] Ly JQM, et al. (2016) Circadian regulation of human cortical excitability. Nat Commun 7:11828. 10.1038/ncomms11828 27339884 PMC4931032

[B51] Marcus DS, Harwell J, Olsen T, Hodge M, Glasser MF, Prior F, Jenkinson M, Laumann T, Curtiss SW, Van Essen DC (2011) Informatics and data mining tools and strategies for the Human Connectome Project. Front Neuroinform 5:4. 10.3389/fninf.2011.00004 21743807 PMC3127103

[B52] Moeller S, Yacoub E, Olman CA, Auerbach E, Strupp J, Harel N, Uğurbil K (2010) Multiband multislice GE-EPI at 7 tesla, with 16-fold acceleration using partial parallel imaging with application to high spatial and temporal whole-brain fMRI. Magn Reson Med 63:1144–1153. 10.1002/mrm.22361 20432285 PMC2906244

[B53] Moore RY, Eichler VB (1972) Loss of a circadian adrenal corticosterone rhythm following suprachiasmatic lesions in the rat. Brain Res 42:201–206. 10.1016/0006-8993(72)90054-6 5047187

[B54] Moore RY, Speh JC (1993) GABA is the principal neurotransmitter of the circadian system. Neurosci Lett 150:112–116. 10.1016/0304-3940(93)90120-A 8097023

[B55] Musiek ES, Holtzman DM (2016) Mechanisms linking circadian clocks, sleep, and neurodegeneration. Science 354:1004–1008. 10.1126/science.aah4968 27885006 PMC5219881

[B56] Muto V, et al. (2016) Local modulation of human brain responses by circadian rhythmicity and sleep debt. Science 353:687–690. 10.1126/science.aad2993 27516598

[B57] Nagano M, Adachi A, Nakahama K-I, Nakamura T, Tamada M, Meyer-Bernstein E, Sehgal A, Shigeyoshi Y (2003) An abrupt shift in the day/night cycle causes desynchrony in the mammalian circadian center. J Neurosci 23:6141–6151. 10.1523/JNEUROSCI.23-14-06141.2003 12853433 PMC6740348

[B58] Nakajima K, Osada T, Ogawa A, Tanaka M, Oka S, Kamagata K, Aoki S, Oshima Y, Tanaka S, Konishi S (2022) A causal role of anterior prefrontal-putamen circuit for response inhibition revealed by transcranial ultrasound stimulation in humans. Cell Rep 40:111197. 10.1016/j.celrep.2022.111197 35977493

[B59] Nakamura K, Brown RA, Narayanan S, Collins DL, Arnold DL (2015) Diurnal fluctuations in brain volume: statistical analyses of MRI from large populations. Neuroimage 118:126–132. 10.1016/j.neuroimage.2015.05.077 26049148

[B60] Nakamura TJ, Nakamura W, Yamazaki S, Kudo T, Cutler T, Colwell CS, Block GD (2011) Age-related decline in circadian output. J Neurosci 31:10201–10205. 10.1523/JNEUROSCI.0451-11.2011 21752996 PMC3155746

[B61] Naseri Kouzehgarani G, Bothwell MY, Gillette MU (2020) Circadian rhythm of redox state regulates membrane excitability in hippocampal CA1 neurons. Eur J Neurosci 51:34–46. 10.1111/ejn.14334 30614107 PMC6609501

[B62] Ogawa A, Osada T, Tanaka M, Kamagata K, Aoki S, Konishi S (2020) Connectivity-based localization of human hypothalamic nuclei in functional images of standard voxel size. Neuroimage 221:117205. 10.1016/j.neuroimage.2020.117205 32735999

[B63] Ogawa A, et al. (2022) Hypothalamic interaction with reward-related regions during subjective evaluation of foods. Neuroimage 264:119744. 10.1016/j.neuroimage.2022.119744 36368500

[B64] Oka S, et al. (2024) Diurnal variation of brain activity in the human suprachiasmatic nucleus. J Neurosci 44:e1730232024. 10.1523/JNEUROSCI.1730-23.2024 38238074 PMC10883613

[B65] Orban C, Kong R, Li J, Chee MWL, Yeo BTT (2020) Time of day is associated with paradoxical reductions in global signal fluctuation and functional connectivity. PLoS Biol 18:e3000602. 10.1371/journal.pbio.3000602 32069275 PMC7028250

[B66] Osada T, Suzuki R, Ogawa A, Tanaka M, Hori M, Aoki S, Tamura Y, Watada H, Kawamori R, Konishi S (2017) Functional subdivisions of the hypothalamus using areal parcellation and their signal changes related to glucose metabolism. Neuroimage 162:1–12. 10.1016/j.neuroimage.2017.08.056 28844890

[B67] Osada T, et al. (2021) Parallel cognitive processing streams in the human prefrontal cortex: parsing the areal-level brain network for response inhibition. Cell Rep 36:109732. 10.1016/j.celrep.2021.109732 34551294

[B68] Osada T, Nakajima K, Shirokoshi T, Ogawa A, Oka S, Kamagata K, Aoki S, Oshima Y, Tanaka S, Konishi S (2024) Multiple insular-prefrontal pathways underlie perception to execution during response inhibition in humans. Nat Commun 15:10380. 10.1038/s41467-024-54564-9 39627197 PMC11615282

[B69] Poppenk J, Evensmoen HR, Moscovitch M, Nadel L (2013) Long-axis specialization of the human hippocampus. Trends Cogn Sci 17:230–240. 10.1016/j.tics.2013.03.005 23597720

[B70] Puig S, et al. (2023) Circadian rhythm disruptions associated with opioid use disorder in synaptic proteomes of human dorsolateral prefrontal cortex and nucleus accumbens. Mol Psychiatry 28:4777–4792. 10.1038/s41380-023-02241-6 37674018 PMC10914630

[B71] Rath MF, Rohde K, Fahrenkrug J, Møller M (2013) Circadian clock components in the rat neocortex: daily dynamics, localization and regulation. Brain Struct Funct 218:551–562. 10.1007/s00429-012-0415-4 22527123

[B72] Rath MF, Rovsing L, Møller M (2014) Circadian oscillators in the mouse brain: molecular clock components in the neocortex and cerebellar cortex. Cell Tissue Res 357:743–755. 10.1007/s00441-014-1878-9 24842045

[B73] Rison NO, Ogawa A, Osada T, Konishi S (2024) Stereotaxic coordinates of human hypothalamic nuclei used for region of interest analyses in functional magnetic resonance imaging. Juntendo Med J 70:129–131. 10.14789/jmj.JMJ24-0009-P PMC11487351 39430209

[B74] Rivera-Rivera LA, Roberts GS, Peret A, Langhough RE, Jonaitis EM, Du L, Field A, Eisenmenger L, Johnson SC, Johnson KM (2024) Unraveling diurnal and technical variability in cerebral hemodynamics from neurovascular 4D-flow MRI. J Cereb Blood Flow Metab 44:1362–1375. 10.1177/0271678X241232190 PMC11342721 38340787

[B75] Robinson EC, Jbabdi S, Glasser MF, Andersson J, Burgess GC, Harms MP, Smith SM, Van Essen DC, Jenkinson M (2014) MSM: a new flexible framework for multimodal surface matching. Neuroimage 100:414–426. 10.1016/j.neuroimage.2014.05.069 24939340 PMC4190319

[B76] Robinson EC, et al. (2018) Multimodal surface matching with higher-order smoothness constraints. Neuroimage 167:453–465. 10.1016/j.neuroimage.2017.10.037 29100940 PMC5991912

[B77] Rosenke M, van Hoof R, van den Hurk J, Grill-Spector K, Goebel R (2021) A probabilistic functional atlas of human occipito-temporal visual cortex. Cereb Cortex 31:603–619. 10.1093/cercor/bhaa246 32968767 PMC7727347

[B78] Ruby NF, Hwang CE, Wessells C, Fernandez F, Zhang P, Sapolsky R, Heller HC (2008) Hippocampal-dependent learning requires a functional circadian system. Proc Natl Acad Sci U S A 105:15593–15598. 10.1073/pnas.0808259105 18832172 PMC2563080

[B79] Salimi-Khorshidi G, Douaud G, Beckmann CF, Glasser MF, Griffanti L, Smith SM (2014) Automatic denoising of functional MRI data: combining independent component analysis and hierarchical fusion of classifiers. Neuroimage 90:449–468. 10.1016/j.neuroimage.2013.11.046 24389422 PMC4019210

[B80] Saper CB, Lu J, Chou TC, Gooley J (2005a) The hypothalamic integrator for circadian rhythms. Trends Neurosci 28:152–157. 10.1016/j.tins.2004.12.009 15749169

[B81] Saper CB, Scammell TE, Lu J (2005b) Hypothalamic regulation of sleep and circadian rhythms. Nature 437:1257–1263. 10.1038/nature04284 16251950

[B82] Schmidt C, et al. (2009) Homeostatic sleep pressure and responses to sustained attention in the suprachiasmatic area. Science 324:516–519. 10.1126/science.1167337 19390047

[B83] Schoonderwoerd RA, et al. (2022) The photobiology of the human circadian clock. Proc Natl Acad Sci U S A 119:e2118803119. 10.1073/pnas.2118803119 35312355 PMC9060497

[B84] Shannon BJ, Dosenbach RA, Su Y, Vlassenko AG, Larson-Prior LJ, Nolan TS, Snyder AZ, Raichle ME (2013) Morning-evening variation in human brain metabolism and memory circuits. J Neurophysiol 109:1444–1456. 10.1152/jn.00651.2012 23197455 PMC3602835

[B85] Smith SM, et al. (2004) Advances in functional and structural MR image analysis and implementation as FSL. Neuroimage 23:208–219. 10.1016/j.neuroimage.2004.07.051 15501092

[B86] Smith SM, et al. (2013) Resting-state fMRI in the Human Connectome Project. Neuroimage 80:144–168. 10.1016/j.neuroimage.2013.05.039 23702415 PMC3720828

[B87] Stephan FK, Zucker I (1972) Circadian rhythms in drinking behavior and locomotor activity of rats are eliminated by hypothalamic lesions. Proc Natl Acad Sci U S A 69:1583–1586. 10.1073/pnas.69.6.1583 4556464 PMC426753

[B88] Suda A, Osada T, Ogawa A, Tanaka M, Kamagata K, Aoki S, Hattori N, Konishi S (2020) Functional organization for response inhibition in the right inferior frontal cortex of individual human brains. Cereb Cortex 30:6325–6335. 10.1093/cercor/bhaa188 32666077 PMC7609925

[B89] Sumova A, Travnicnkova Z, Peterst R, Schwartzt WJ, Illnerova H (1995) The rat suprachiasmatic nucleus is a clock for all seasons. Proc Natl Acad Sci U S A 92:7754–7758. 10.1073/pnas.92.17.7754 7644490 PMC41224

[B90] Vandewalle G, et al. (2010) Spectral quality of light modulates emotional brain responses in humans. Proc Natl Acad Sci U S A 107:19549–19554. 10.1073/pnas.1010180107 20974959 PMC2984196

[B91] Van Essen DC (2005) A population-average, landmark- and surface-based (PALS) atlas of human cerebral cortex. Neuroimage 28:635–662. 10.1016/j.neuroimage.2005.06.058 16172003

[B92] Welsh DK, Logothetis DE, Meister M, Reppert SM (1995) Individual neurons dissociated from rat suprachiasmatic nucleus express independently phased circadian firing rhythms. Neuron 14:697–706. 10.1016/0896-6273(95)90214-7 7718233

[B93] Welsh DK, Takahashi JS, Kay SA (2010) Suprachiasmatic nucleus: cell autonomy and network properties. Annu Rev Physiol 72:551–577. 10.1146/annurev-physiol-021909-135919 20148688 PMC3758475

[B94] Yamaguchi S, Isejima H, Matsuo T, Okura R, Yagita K, Kobayashi M, Okamura H (2003) Synchronization of cellular clocks in the suprachiasmatic nucleus. Science 302:1408–1412. 10.1126/science.1089287 14631044

[B95] Yang S, Wang K, Valladares O, Hannenhalli S, Bucan M (2007) Genome-wide expression profiling and bioinformatics analysis of diurnally regulated genes in the mouse prefrontal cortex. Genome Biol 8:R247. 10.1186/gb-2007-8-11-r247 18028544 PMC2258187

[B96] Yeo BTT, et al. (2011) The organization of the human cerebral cortex estimated by intrinsic functional connectivity. J Neurophysiol 106:1125–1165. 10.1152/jn.00338.2011 21653723 PMC3174820

[B97] Zhang S, Wang W, Zhornitsky S, Li CSR (2018) Resting state functional connectivity of the lateral and medial hypothalamus in cocaine dependence: an exploratory study. Front Psychiatry 9:344. 10.3389/fpsyt.2018.00344 30100886 PMC6072838

